# Variability in the Aerobic Fitness-Related Dependence on Respiratory Processes During Muscle Work Is Associated With the ACE-I/D Genotype

**DOI:** 10.3389/fspor.2022.814974

**Published:** 2022-05-19

**Authors:** Benedikt Gasser, Annika Frei, David Niederseer, Silvio Catuogno, Walter O. Frey, Martin Flück

**Affiliations:** ^1^Departement für Bewegung und Sport, Universität Basel, Basel, Switzerland; ^2^Laboratory for Muscle Plasticity, University of Zurich, Zurich, Switzerland; ^3^Department of Cardiology, University Hospital Zurich, University of Zurich, Zurich, Switzerland; ^4^Swiss Olympic Medical Center, Balgrist University Hospital, Zurich, Switzerland; ^5^Swiss Federal Institute of Sport, Macolin, Switzerland

**Keywords:** pathway of oxygen, oxygen saturation, hemoglobin, ventilation, cardiac output, exhaustion, aerobic metabolism

## Abstract

**Background:**

The efficiency of aerobic energy provision to working skeletal muscle is affected by aerobic fitness and a prominent insertion/deletion polymorphism in the angiotensin-converting enzyme (ACE-I/D) gene for the major modulator of tissue perfusion. We assessed whether variability in the fitness state is dependent on the contribution of multiple aspects of oxygen transport to the development of muscle power, and the respective control coefficients, are associated with the ACE-I/D genotype.

**Methods:**

Twenty-five women and 19 men completed a ramp test of cycling exercise to exhaustion during which serial steps of oxygen transport [oxygen uptake (L O_2_ min^−1^) (VO_2_), minute ventilation in (L min^−1^) (VE), cardiac output in equivalents of L min^−1^ (Q), arterial oxygen saturation (SpO_2_), muscle oxygen saturation (SmO_2_), and total hemoglobin concentration (g dL^−1^) (THb) in *Musculus vastus lateralis* and *Musculus gastrocnemius*, respiration exchange ratio (RER)], blood lactate and glucose concentration, were continuously monitored. The contribution/reliance of power output (PO) on the parameters of oxygen transport was estimated based on the slopes in Pearson's moment correlations (|*r*| > 0.65, *p* < 0.05) vs. power values over the work phase of the ramp test, and for respective fractional changes per time (defining control coefficients) over the rest, work, and recovery phase of the ramp test. Associations of variability in slopes and control coefficients with the genotype and aerobic fitness were evaluated with ANOVA.

**Results:**

All parameters characterizing aspects of the pathway of oxygen, except THb, presented strong linear relationships [(|*r*| > 0.70) to PO]. Metabolic efficiency was 30% higher in the aerobically fit subjects [peak oxygen uptake (mL O_2_ min^−1^) (VO_2_peak) ≥ 50 ml min^−1^ kg^−1^], and energy expenditure at rest was associated with the fitness state × ACE-I/D genotype, being highest in the fit non-carriers of the ACE D-allele. For VO_2_, VE, and RER the power-related slopes of linear relationships during work demonstrated an association with aerobic fitness, being 30–40% steeper in the aerobically fit than unfit subjects. For VE the power-related slope also demonstrated an association with the ACE-I/D genotype. For increasing deficit in muscle oxygen saturation (DSmO_2_) in *Musculus vastus lateralis* (DSmO_2_ Vas), the power-related slope was associated with the interaction between aerobic fitness × ACE-I/D genotype.

**Conclusion:**

Local and systemic aspects of aerobic energy provision stand under influence of the fitness state and ACE-I/D genotype. This especially concerns the association with the index of the muscle's mitochondrial respiration (SmO_2_) which compares to the genetic influences of endurance training.

## Introduction

Endurance performance depends on the capacity for aerobic energy provision. Thereby, linear connectivity is implied between the mechanical work being produced and the upstream oxygen delivery system (Hoppeler and Weibel, 2000). Differences in the metabolic efficiency and economy during submaximal endurance exercises are reflected by ameliorations of multiple aspects of the coupled organismal processes that connect pulmonary oxygen uptake *via* hemoglobin-mediated oxygen transport to aerobic generation and use of ATP in working skeletal muscle (reviewed in Hoppeler and Weibel, 2000). Thereby the contribution of individual parameters to alterations in aerobic metabolism with activity-induced energy expenditure is indicated by control coefficients that refer to the relationship between increments of change (Veech and Fell, 1996; Suarez and Moyes, 2012). The structure and function of the oxygen delivery system to skeletal muscle are thereby built under influence of certain genetic factors, which affect the metabolic efficiency of muscle contraction during exercise (Montgomery and Brull, 2000).

One factor contributing to inter-individual differences in oxygen transport during exercise, and thus potentially metabolic efficiency, is vascularization and, in consequence, the gene for angiotensin-converting enzyme (ACE) (Kamiya et al., 1990; Montgomery and Brull, 2000; Dékány et al., 2006; Puthucheary et al., 2011; Valdivieso et al., 2017). Thereby, the presence of a 287-basepair insertion (the I-allele) is associated with silencing of the expression of the encoded ACE peptidase (Mathes et al., 2015; van Ginkel et al., 2016).

In consequence, there is reduced processing of the major myogenic regulator of smooth and cardiac muscle, the octapeptide angiotensin II, and the degradation of the vasodilative kinin peptides (Peach, 1981; Valdivieso et al., 2017). ACE deletion allele carriers are typically characterized by a lowered peripheral blood flow during exercise due to a higher peripheral vascular resistance (Mathes et al., 2015; Valdivieso et al., 2017). Furthermore, the ACE D-allele is associated with left ventricular hypertrophy and consequently elevated cardiac output, especially with physical training (Hagberg et al., 2002; Hernández et al., 2003; Bahramali et al., 2016). As well, the ACE-I/D genotype is associated with variability in maximal oxygen uptake, minute ventilation, and arterial oxygen saturation, especially under the extreme condition of high altitude (Woods et al., 2002; Patel et al., 2003; Williams et al., 2017).

Additionally, and importantly, the ACE-I/D gene polymorphism is interdependently interacting with physical activity levels regarding influences on the metabolic processes that are associated with muscle performance during exercise. This manifests, for example, on a structural level in peripheral skeletal muscle in differences for capillarization, mitochondrial volume density (Vaughan et al., 2013; Valdivieso et al., 2017), and the connected variability in lipid, glucose, and amino acid metabolism between ACE-I/D genotypes (Valdivieso et al., 2017). In summary, the reported influence of the ACE-I/D gene polymorphism on metabolic efficiency and economy (Montgomery and Brull, 2000; Woods et al., 2002) notably the better trainability of endurance-related traits of aerobic performance in subjects with the I-allele of ACE involves multiple aspects of oxygen delivery from the pulmonary level to contracting skeletal muscle (Hernández et al., 2003; Flueck et al., 2010; Puthucheary et al., 2011; Vaughan et al., 2013; Valdivieso et al., 2017).

The quantitative influence of the ACE-I/D polymorphism on the different aspects of oxygen use and delivery and mechanical output, as well as their interdependence during endurance-type exercise is not known. We hypothesized that the ACE-I/D genotype affects the contribution of the different aspects of systemic oxygen delivery to the production of muscle power during submaximal exercise. We specifically assumed that the dependencies of power output (PO) on aerobic metabolism, as estimated based on the slopes of the respective linear relationships between raw values and their fractional changes per time (which reveal the control coefficients) to PO, would be enhanced in aerobically fit non-carriers of the ACE D-allele during the different phases of cycling type exercise to exhaustion, when power-related slopes for cardiac output were expected to demonstrate an increased contribution to performance in D-allele.

## Methods

### Subjects

A total of 44 healthy subjects (19 men and 25 women) performed a ramp test of cycling exercise to exhaustion. Subjects had to meet the inclusion criteria (good health, age of 20–70 years, absence of cardiovascular diseases) (Table 1).

**Table 1:** Subjects characteristics—Mean ± SD and interaction effects between aerobic fitness × ACE-I/D genotype on physiological characteristics.

		**Age**	**Body mass**	**Height**	**BMI**	**VO_**2**_peak**	**Relative VO_**2**_peak**	**PPO**
	***n***	**Years**	**kg**	**m**	**kg m^**−2**^**	**mL·min^−1^**	**mL·min^−1^·kg^−1^**	**W**
Unfit noD	8	37.1 ± 13.9	74.9 ± 10.6	171.5 ± 4.5	25.4 ± 3.3	2,702.2 ± 226.4	37.1 ± 9.6	243.6 ± 45.3
Unfit D	19	34.7 ± 14.4	66.9 ± 9.7	170.5 ± 8.4	22.9 ± 1.8	2,639.9 ± 146.9	39.2 ± 6.8	242.5 ± 66.9
Fit noD	3	27.9 ± 1.1	76.6 ± 0.6	177.3 ± 3.5	24.4 ± 0.8	4,172.6 ± 369.7	54.5 ± 3.5	400.0 ± 26.5
Fit-D	14	31.7 ± 7.0	68.3 ± 10.5	176.3 ± 8.5	21.8 ± 1.9	3,819.8 ± 171.2	56.0 ± 4.2	354.1 ± 52.4
Fitness state	*p*	0.185	0.685	0.055	0.201	**<**0.001	**<**0.001	**<**0.001
	h2	0.044	0.004	0.089	0.041	0.424	0.537	0.483
Genotype	*p*	0.882	0.037	0.742	0.003	0.401	0.475	0.289
	h2	0.001	0.105	0.003	0.196	0.018	0.013	0.028
Fitness state × genotype	*p*	0.498	0.959	0.986	0.992	0.556	0.904	0.313
	h2	0.012	<0.001	<0.001	<0.001	0.009	<0.001	0.025

*Bold underlined values met the criteria of p < 0.05. ANOVA with post-hoc test of least significant difference. N = 44*.

### Experimental Design

The investigation had a cross-sectional design in which healthy subjects reported resting in the laboratory and performing a bout of pedaling exercise that ramped intensity from low intensity to an intensity that produced voluntary exhaustion to conduct cardio-respiratory measurements and characterize aerobic fitness based on peak oxygen uptake (mL O_2_ min^−1^) (VO_2_peak) and peak aerobic PO. During the entire duration of the ramp test PO, muscle oxygen saturation (SmO_2_), and total hemoglobin concentration (g dL^−1^) (THb) in a knee extensor muscle (*musculus vastus lateralis*) and a plantar flexor muscle (*musculus gastrocnemius*), were monitored based on near-infrared spectroscopy to estimate mitochondrial respiration (Ryan et al., 2014) and perfusion, respectively (Gasser et al., 2021). Heart rate, systolic and diastolic blood pressure, and the concentration of glucose and lactate were measured every second minute commencing at rest and continuing into the recovery phase after pedaling. As the average age of participants was for all subsamples (see Table 1) around 30 years in accordance with the guidelines of the American Society of Sport Medicine, subjects were assigned as being aerobically fit or unfit based on whether their respective VO_2_peak met the criteria of being above or below 50 mL·min−1 kg−1, whereby this cut-off was crosschecked with other thresholds (Supplementary Table 1). (Valdivieso et al., 2017; ACSM, 2021). Genotyping was performed subsequently. The effects of “ACE-I/D genotype” × “aerobic fitness” status on the strength of the linear relationships of parameters reflecting the systemic pathway of oxygen vs. power were statistically assessed. The statistical inspection was based on the hypothesis that a dominant model of carrying the D-allele would identify expected and novel relationships.

### Ramp-Incremental Exercise to Exhaustion

The test protocol was conducted in an air-conditioned laboratory at a standardized temperature of 20°C according to a modified version of a published protocol by Swiss Olympic (Whipp et al., 1981). In brief, the test started with 3 min of rest, when subjects sat still on the cycle ergometer while maintaining a normal breathing pattern without warming up subsequently, subjects started pedaling at an initial power (75 W for women and 100 W for men). Target power was increased every 20 s (18 W·min^−1^ for women and 30 W·min^−1^ for men). The subjects were asked to keep a constant self-chosen pedal cadence between 70 and 100 rpm throughout the test. During the entire duration of the exercise test, subjects were advised and supervised by an experienced sport scientist. The test was stopped when the subjects experienced volitional exhaustion and/or were not able to maintain the target pedal cadence and power output. Subsequently, recordings continued during a period of eight min, when subjects rested in a seated position on the cycle ergometer. Cardiorespiratory parameters were continuously monitored with a Metalyzer 3B-R2 device (Cortex, Leipzig, Germany) and this was accompanied by the assessment of blood pressure (SunTech Medical, North Carolina, USA) and heart rate (suunto, Vantaa, Finland), SpO_2_ (arterial oxygen saturation), heart rate, systolic and diastolic blood pressure, and minute ventilation in (L min^−1^) (VE). Cardiac output was estimated based on the assessed parameters according to Liljestrand and Zander (Koenig et al., 2015). Local measurements of SmO_2_ and THb were carried out from the muscle with near-infrared spectroscopy. Measurements commenced with the start of pedaling and were stopped 8 min into recovery from exercise. The rate of perceived exertion was assessed each second min with the Borg scale (Borg, 1982).

### Measurement of Blood Glucose and Lactate

Two 20 μl-aliquots of capillary blood were drawn from the ear lobe and subjected to the quantification of glucose using the OneTouch^®^ Vita™ Blood Glucose Monitoring System (Lifescan, Milpitas, CA, USA) or lactate using a Biosen C-line analyzer (EKF Diagnostic GmbH, Barleben, Germany). Cardiovascular measurements during interval-type of pedaling exercise was monitored with a pulse belt (Suunto, Vantaa, Finland) and with a pulsoxymeter (Sonomed GMBH, Volketswil, Zürich). Systolic and diastolic blood pressures were manually assessed.

### Near-Infrared Spectroscopy

A portable muscle oxygen monitor (Moxy, Fortiori Design LLC, Hutchinson, Minnesota, United States) was used to measure muscle oxygen saturation and hemoglobin non-invasively as previously described by us and others (Ryan et al., 2014; Design, 2015; Vaughan et al., 2016; Jones et al., 2017; Perrey and Ferrari, 2018). Pulmonary gas exchange (oxygen uptake) was measured through a breath-by-breath spiroergometry system after initial proper calibration (MetaLyzer^®^3B-R2, CORTEX Biophysics, Leipzig, Germany). VO_2_peak and peak PO were defined as the last achieved, and peak, values before exhaustion manifested. The device uses four different light sources covering wavelengths in the range of 630–850 nm and a modified Beer-Lambert law to perform measurements of SmO_2_. The sensor was placed on the lower third of *m. vastus lateralis* in the middle of the muscle belly on the left leg of the subjects. Prior to the placement, the skin site was shaved using a disposable razor (Gallant, Dynarex, Orangeburg, USA) and cleaned with an alcohol swab (Webcol™, Covidien™, Dublin, Ireland). The sensor was attached using the recommended tape (Moxy Adhesive Attachments, Fortiori Design LLC, Minnesota, United States). To protect the NIRS device from ambient light it was covered with an adhesive non-woven fabric (Hypafix^®^, BSNmedical, Hamburg, Germany).

### Genotyping

Typing of the ACE-I/D gene polymorphism was carried out with modifications of the described protocol (Vaughan et al., 2016). In brief, genomic material was extracted from fresh buccal swabs through the use of a commercially available protocol that comprised the degradation of ribonucleic acid and proteins based on RNAse and proteinase K and a last step of filtration with a QIAamp Mini spin column (QIAamp^®^ DNA Mini Kit, Qiagen, Hilden, Germany). Two microliters of the resulting eluate (150 μl in total) were subjected to a polymerase chain reaction in 48-well plates with a specific combination of primer sets followed by high-resolution melt analysis with a real-time PCR system (Eco™, illumina^®^, San Diego, United States) as described (Valdivieso et al., 2017). The primer mix for the detection of the I-allele specific 66 bp amplicon contained the primer ACE2 (5′-tgggattacaggcgtgatacag-3′) and the primer ACE3 (5′atttcagagctggaataaaatt-3′). The primer mix for the detection of the D-allele specific 83 bp amplicon contained primers ACE1 (5′-catcctttctcccatttctc-3′) and ACE3 (5′atttcagagctggaataaaatt-3′). Genotype analysis was carried out using genetic variation analysis software (EcoStudy Version 5.0, Illumina^®^, San Diego, United States).

### Data Handling and Analysis

**Figure 1:**
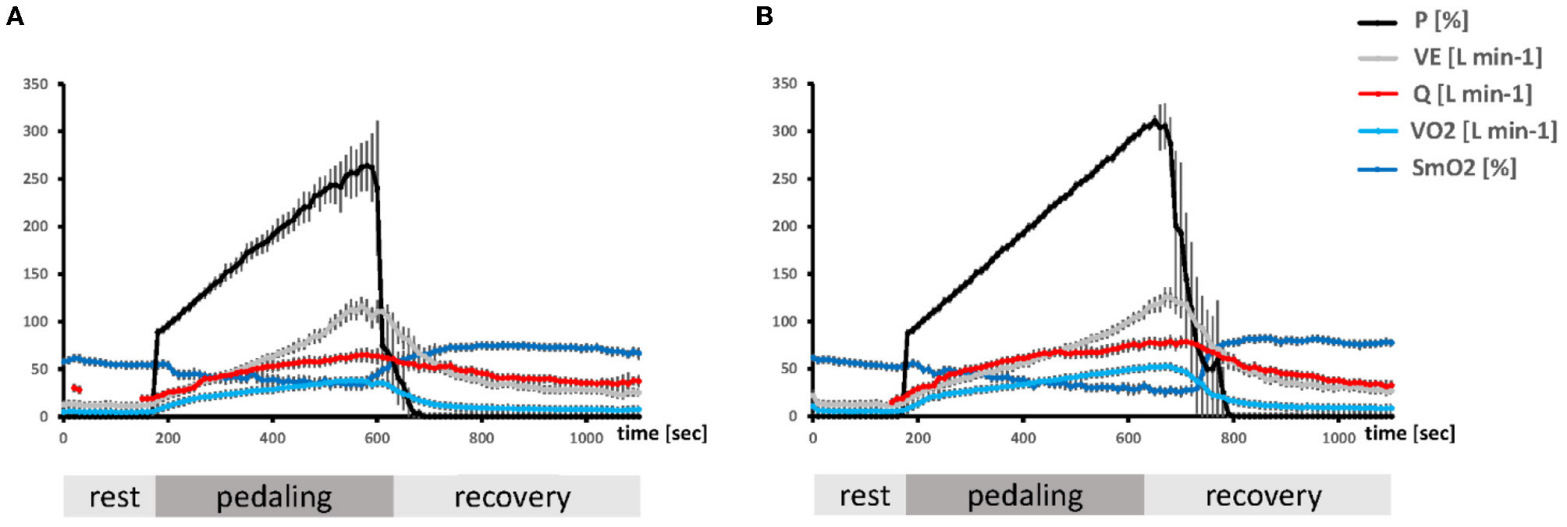
Timeline of the average metabolic alterations during the ramp test. Composite line graphs visualizing the mean + SE over 10-s-intervals of the ramp exercise test for selected parameters characterizing aerobic metabolism and PO in unfit **(A)** and aerobically fit **(B)** subjects. Values during the pedaling phase were scaled to the respective average duration during which subjects were able to resist voluntary exhaustion. The Y-axes indicate the numerical values for each parameter.

Each data set being continuously recorded during the ramp test, i.e., PO, VO_2_, ventilation, heart rate, systolic blood pressure, diastolic blood pressure, arterial oxygen saturation, VCO_2_ and respiration exchange rate, and the NIRS-based data series, was imported and assembled in MS-Excel (MS Office, Kildare, Ireland), synchronized and inspected to verify the integrity of the data and correct inconsistent values. An example of the course of recorded values during the ramp test is shown in Figure 1.

Energy consumption and mechanical /aerobic efficiency were calculated based on the values of oxygen uptake (L O_2_ min^−1^) (VO_2_) and respiration exchange ratio (RER) measured during the ramp test, essentially as described (Jabbour and Majed, 2019) with the following formulas: Energy (E) = (4.94 ^*^ RER + 16.04) × VO_2_net; VO_2_net = VO_2_-VO_2_rest; where VO_2_ was expressed in ml s^−1^. Furthermore, efficiency was set to W/E.

Average values and standard error for selected parameters characterizing aerobic metabolism and PO for the group of unfit and fit (B) subjects were calculated over 10-s-intervals of the ramp exercise test in MS-Excel. For both groups, the values for the time during the pedaling phase were scaled to the respective average duration during which fit subjects were able to resist voluntary exhaustion.

The “accrued oxygen deficit” and the “accrued hemoglobin” during exercise were calculated from the area under the time curve for the recorded SmO_2_ and THb data sets during the exercise. For the assessment of control rates, the sampled data of each participant were analyzed for the values of the increments of change per time in the five phases of metabolic rate [rest (0–180 s), transition rest to exercise (180–206 s), exercise (until the cessation of pedaling), transition exercise to recovery (first 36 s after the end of exercise) and recovery (436 s after cessation of exercise)]. Subsequently, the respective mean value of the increments for the parameters of the pathway of oxygen and PO was calculated for each phase and participant.

### Statistics

Pearson's moment correlations were carried out with MS-Excel to determine the degree of linear relationships between the raw values for parameters of the pathway of oxygen vs. PO and oxygen uptake during the work phase, and for the respective fractional changes per time per fractional changes of power (defining control coefficients) over the five metabolic phases of the ramp-incremental exercise, i.e., rest, work, recovery and the transition phases in between. The resulting *r*- and *p*-values, and slopes (indicative of the dependence), were exported and verified. Linear relationships were considered for cases where |*r*| > 0.65 and *p* < 0.05 and the network of correlations over the work phase were displayed using Cytoscape software (3.5.0) as described (Shannon et al., 2003). For the display of the difference in linear relationships for the respective fractional changes per time vs. PO over the different metabolic phases, the calculated mean values were normalized to the mean values during exercise and plotted as median and standard error. For the ramp exercise to exhaustion, univariate (ANOVA) and multivariate analyses of variance (MANOVA) with *post-hoc* test for the least significant difference were run to identify effects of the factors “aerobic fitness” × “ACE-I/D” genotype, on the *r*-values and slopes of linear correlations during ramp exercise. Genotype-specific differences were calculated based on a dominant genetic model for the ACE D-allele (I/I vs. I/D vs. D/D). The Shapiro-Wilks test was used to analyze normal distribution in the measured and, respectively, derived parameters [DSmO_2_ in m. vastus lateralis (DSmO_2_ VAS), DSmO_2_ in m. gastrocnemius (DSmO_2_ GAS), THb in m. vastus lateralis (THb VAS), THb in m. gastrocnemius (THb GAS), VO_2_, SpO_2_, cardiac output in equivalents of L min^−1^ (Q), VO_2_ per kg, VE, RER] over power, whereby the hypothesis of normal distribution could not be rejected for the majority of measurements except VO_2_ per kg, VO_2_, and RER. Furthermore, variance homogeneity for sub-samples was analyzed with Levene test, whereby only for VO_2_/kg variance inhomogeneity was deduced. Differences with a *p* < 0.05 were considered as significant. No adjustments for multiple comparisons were directly performed as the factors analyzed were connected and changed the result concomitantly as a consequence of known biological connection; however, hints are made in the respective tables (see e.g., Table 1). Values were displayed in box-whisker plots in MS-Excel. Concerning sample size, it is to mention that a prospective power analysis based on the reported associations of the ACE-I/D genotype indicated that the statistical significance of hypothesized interaction effects between ACE-I/D genotype × fitness state would be reached with the recruitment of 6–10 subjects (α = 0.05, β = 0.8; for details see Supplementary Table 2). Linear relationships between parameters were displayed using Cytoscape software (3.5.0) as described (Shannon et al., 2003). Statistical analyses were carried out with MS-Excel (Microsoft Office Professional Plus 2016, Kildare, Ireland) and the Statistical Package for the Social Sciences (SPSS version 23, IBM, Armonk, United States).

## Results

### Subjects' Characteristics

Table 1 summarizes the biological characteristics of the 44 studied volunteers. No significant differences concerning average age could be detected between the subjects taxed as fit and unfit (*p* = 0.259). The subjects being declared as aerobically fit demonstrated on average a 17.2 mlO_2_ min^−1^ kg^−1^ higher specific VO_2_peak and 119.4 Watts higher peak aerobic PO than the unfit subjects. Body mass and BMI did not differ between the aerobically fit and unfit subjects. The ACE-I/D genotype was not associated with differences in VO_2_peak or PPO independent of whether it was assessed as a single effect or interaction effect with the aerobic fitness state. The body mass and BMI were lower in carriers compared to non-carriers of the D-allele.

Body mass-related parameters, i.e., body mass (*p* < 0.001), BMI (*p* = 0.002), height (*p* = 0.001), and peak power output (PPO) (*p* < 0.001), demonstrated an influence of sex, which disappeared when the interaction with the aerobic fitness state and/or ACE-I/D genotype was considered.

### Metabolic Characteristics of the Challenge of Incremental Pedaling Exercise

The exercise test was characterized by phases of rest, a ramp of incremental pedaling, and subsequent recovery, whereby the two phases of transition (from rest to exercise and from exercise to recovery) produced substantial alterations in main parameters of the pathway of oxygen, i.e., VO_2_, VE, Q, SmO_2_ (Figure 1).

The values for VO_2_, VE, and SmO_2_, were fairly stable during the first phase of rest and were more or less steadily affected with the onset of contraction up to relatively extreme values when voluntary exhaustion manifested. The values for all parameters, recovered to, or above baseline values, with the cessation of exercise. THb in *m. vastus lateralis* showed a considerably lower degree of change with the onset of contraction, decreasing on average by 0.10 g dL^−1^, i.e., 12.50 ± 0.41 g dL^−1^ vs. 12.59 ± 0.35 g dL^−1^ (*p* = 0.0023).

### Association of Metabolic Alterations With Fitness and the ACE-I/D Genotype

At rest, energy expenditure was 30.4% higher in aerobically fit than unfit subjects. This was due to a concomitantly 29.4% elevated VO_2_ (*p* = 0.0001) when RER was indifferent with respect to the fitness state (*p* = 0.237). RER at rest was affected by the ACE-I/D genotype, where 7.8% higher values (*p* = 0.0081) were observed in carriers than non-carriers of the D-allele, whereby both the unfit and the aerobically fit subjects demonstrated this trend (Table 2). Energy expenditure at rest, as well, demonstrated an interaction effect of the fitness state and ACE-I/D genotype.

**Table 2:** Summary of the effects of aerobic fitness and ACE-I/D genotype on oxygen dependent energy metabolism at rest.

**Status**		**VO_**2**_ rest**	**RER rest**	**E rest**
Unfit noD		0.298 ± 0.021	0.843 ± 0.022	100.327 ± 7.352
Unfit D		0.335 ± 0.016	0.892 ± 0.010	114.205 ± 4.485
Fit noD		0.499 ± 0.036	0.828 ± 0.038	167.401 ± 13.347
Fit D		0.403 ± 0.023	0.921 ± 0.025	138.482 ± 8.453
Fitness	*p*	<0.001	0.797	<0.001
	h2	0.3883	0.002	0.3741
Genotype	*p*	0.279	0.009	0.426
	h2	0.029	0.158	0.016
Fitness × genotype	*p*	0.016	0.4	0.027
	h2	0.136	0.018	0.116
NoD fit vs. unfit		<0.001	0.741	<0.001
D fit vs. unfit		<0.001	0.237	0.008
Unfit noD vs. D		0.208	0.094	0.186
Fit noD vs. D		0.037	0.037	0.071

*Effects, which are underlined in bold, were deemed significant. ANOVA with post-hoc test of least significant difference was performed. N = 44. Definitions are introduced in the list of abbreviations*.

Table 3 reports the interaction effects of exercise time, aerobic fitness, and ACE-I/D genotype on the assessed cardiovascular parameters. Aside systolic and diastolic blood pressures, cardiac output, and RPE, the blood lactate concentration was affected by the exercise time. Blood lactate and glucose concentration were—in addition to systolic blood pressure and cardiac output—affected by the fitness state. All metabolic parameters, except diastolic blood pressure, demonstrated an association with the fitness state × genotype.

**Table 3:** Main effects on cardiovascular parameters during ramp exercise.

						**Blood pressure**		
**Factors**		**RPE**	**Lactate**	**Glucose**	**Heart rate**	**Systolic**	**Diastolic**	**Cardiac output**
		**(BORG)**	**(mM)**	**(mM)**	**(bpm)**	**(mm Hg)**	**(mm Hg)**	**(mm Hg × mm Hg^**−1**^ × bpm)**
Time	*p*-value	**<**0.001	**<**0.001	0.916	**<**0.001	**<**0.001	0.248	**<**0.001
	h2	0.875	0.754	0.014	0.813	0.516	0.047	0.711
Fitness_state	*p*-value	0.376	0.042	0.006	0.562	**<**0.001	0.121	**<**0.001
	h2	0.004	0.022	0.04	0.002	0.067	0.013	0.08
Fitness_state * time	*p*-value	0.359	0.174	0.588	0.957	0.999	0.288	0.301
	h2	0.041	0.054	0.03	0.011	0.003	0.045	0.044
Genotype	*p*-value	0.454	0.238	0.925	0.995	0.023	0.167	0.005
	h2	0.003	0.008	0	0	0.028	0.01	0.043
Genotype * time	*p*-value	0.254	0.807	0.934	0.98	0.842	0.457	0.67
	h2	0.047	0.02	0.013	0.008	0.018	0.035	0.026
Fitness_state * genotype	*p*-value	**<**0.001	0.07	0.006	0.005	0.023	0.38	**<**0.001
	h2	0.081	0.018	0.04	0.042	0.028	0.004	0.077
Fitness_state * genotype * time	*p*-value	0.951	0.814	0.755	0.953	0.511	0.731	0.22
	h2	0.009	0.016	0.018	0.009	0.028	0.019	0.043

*Effects, which are underlined in bold, were deemed significant. Significance levels are presented without correction for multiple comparisons—based on physiological reasoning a correction factor of 3 could be stated (first: lactate, glucose/second: heart rate, systolic blood pressure, cardiac output, RPE/third: diastolic blood pressure). Repeated-measures ANOVA with post-hoc test of least significant difference for the factors exercise time × fitness state × ACE-I/D genotype (i.e., D-allele) were conducted. N = 44*.

**Figure 2:**
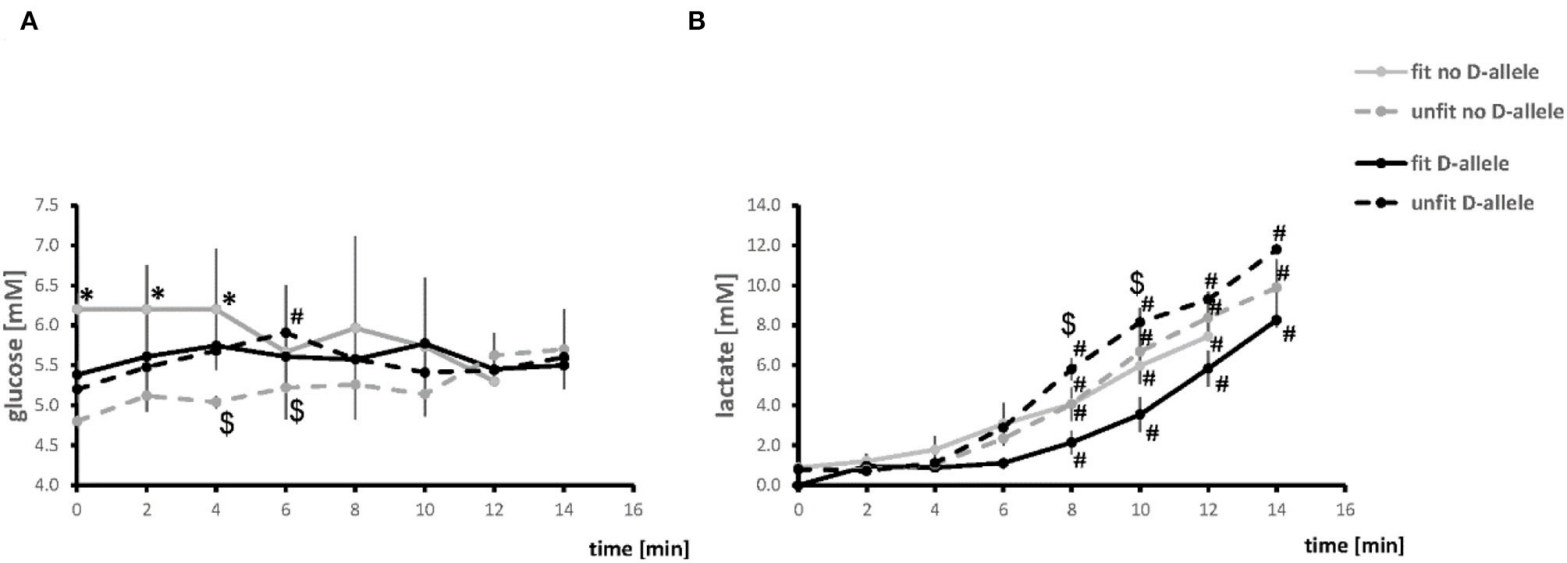
Timeline of alterations of blood metabolites during the pedaling phase of exercise test. Line graph visualizing the mean values of glucose **(A)** and lactate **(B)** concentration in blood during the pedaling phase of exercise. ^#^*p* < 0.05 vs. 0 min, *p < 0.05 vs. unfit, same genotype. ^$^*p* < 0.05 vs. other genotype, same fitness state. Note, the time to exhaustion was two min shorter for the fit D-allele non-carriers, because the all-male group had a steeper ramp protocol.

Figure 2 reports the values of serum glucose and lactate concentration during the pedaling phase of exercise. The blood lactate concentration increased in all combinations of genotype × fitness state starting 8 min into the exercise. For unfit subjects this increase was higher in carriers than non-carriers of the D-allele. The concentration of glucose in blood plasma was higher in the fit than unfit non-carriers of the D-allele up to 4 min of exercise and then declined. In unfit D-allele carriers, glucose increased 6 min into the exercise.

The metabolic efficiency at an intensity corresponding to 100% of the peak aerobic PO demonstrated an influence of the fitness state (*p* = 0.036, h2 = 0.106), but not ACE-I/D genotype (*p* = 0.310), being higher in the fit than unfit subjects (0.348 vs. 0.294).

### Linear Relationships Between Metabolic Parameters and Power Output During the Work Phase of Exercise Test

**Figure 3:**
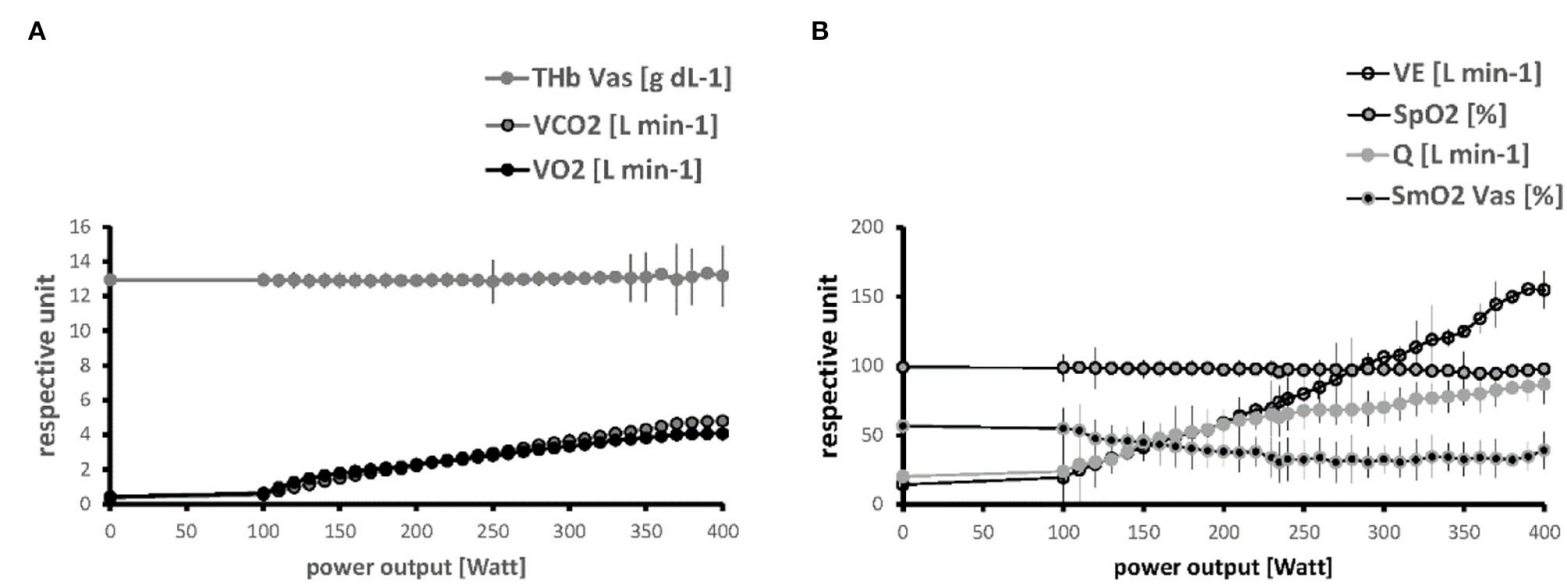
Linearity of relationships between metabolic parameters and power output. Line graphs visualizing the progression of values for parameters of the pathway of oxygen respective to PO during the work phase of the ramp test, for THb, VCO_2_ and VO_2_ **(A)** and VE, SmO_2_, SpO_2_ and Q **(B)**. Values are shown up to a maxima of 400 Watts when most subjects ceased pedaling. Symbols and vertical lines indicate mean + SD of values at an imposed PO in the studied 19 male subjects.

Figure 3 depicts the linear relationships between the average values for the parameters of the systemic pathway of oxygen vs. PO. Steep increases respective to the generated PO were noted (in order of their relative magnitude of change) for VE, Q, VO_2_, and RER, when SmO_2_ in *m. vastus lateralis* and *m. gastrocnemius* demonstrated reductions due to a increasing deficit in muscle oxygen saturation with an increase in the externally imposed PO. THb in *m. vastus lateralis* remained fairly stable, though increasing toward the end of the work phase.

Supplementary Figure 1 provides an overview over the observed linear relationship between the parameters being assessed during the incremental ramp exercise test. Strong correlations passing the threshold of |*r*| > 0.7 existed between PO and VO_2_ and essentially all the parameters characterizing the pathway of oxygen, except for THb.

### Aerobic Fitness and ACE-I/D Genotype Affect Linear Relationships Between Metabolic Parameters and Power Output

During the pedaling exercise, few linear relationships between PO and systemic characteristics of parameters of the pathway of oxygen, were affected by aerobic fitness and its interaction with the -I/D genotype (Table 4). The slopes of the linear relationships vs. PO during the work phase of the exercise test demonstrated an association with aerobic fitness for VO_2_, VE, and RER, when Q demonstrated a trend for such an association (*p* = 0.076). The slope of VE vs. PO demonstrated an association with the ACE-I/D genotype. Interaction effects were resolved between aerobic fitness × ACE-I/D genotype for the power-related slope of DSmO_2_ in *m. vastus lateralis*, when SpO_2_ and the respective slope for THb in *m. vastus lateralis* demonstrated a trend for such an interaction (*p* = 0.074 and 0.053, respectively).

**Table 4:** Association of fitness state × genotype with the slopes between metabolic parameters and power output.

	**Aerobic fitness**		**Genotype**		**Aerobic fitness** **×genotype**	
	***p*-value**	**h2**	***p*-value**	**h2**	**p*-*value**	**h2**
VO_2_	0.022	0.125	0.465	0.013	0.476	0.013
VE	0.021	0.127	0.041	0.1	0.431	0.016
Q	0.076	0.077	0.189	0.043	0.202	0.04
DSpO_2_	0.801	0.002	0.811	0.002	0.074	0.084
DSmO_2_ Vas	0.393	0.018	0.08	0.075	0.016	0.136
DSmO_2_ Gas	0.48	0.013	0.391	0.018	0.115	0.061
THb Vas	0.136	0.055	0.992	0	0.053	0.09
THb Gas	0.769	0.002	0.123	0.058	0.297	0.027
RER	0.002	0.217	0.695	0.004	0.624	0.006

*List of the significance (p) and effect size (h2) of the interaction effects between aerobic fitness × ACE-I/D genotype on the slope of correlations between parameters of the pathway of oxygen and power output during the pedaling phase of the exercise test. Bold underlined values met the criteria of p < 0.05. N = 44. DSpO_2_ (deficit in arterial oxygen saturation)*.

The slope of the linear relationship for body mass-related VO_2_ vs. power, alone, but not absolute VO_2_ (*p* = 0.240), demonstrated an association with the sex (*p* < 0.001), which disappeared when the interaction with the aerobic fitness state (*p* = 0.406) and/or ACE-I/D genotype (*p* = 0.842) was considered.

At the *post-hoc* level, the values for the power-related slopes of VO_2_, VE, and RER were higher in aerobically fit than unfit subjects, when a trend for such a difference was observed for Q (**Figure 5**). The observed interaction effects of fitness × genotype concerned, and extended to, a reduced slope for power-related linear relationship between VE, DSmO_2_ in *m. vastus lateralis*, and *m. gastrocnemius*, as well as THb in either muscle for unfit subjects carrying the D-allele compared to non-carriers of the D-allele.

### Relationships Between the Control Coefficients for Aspects of the Oxygen Pathway vs. Power

For the main parameters characterizing the systemic pathway of oxygen, strong correlations (i.e., |*r*| > 0.85) were identified between the respective mean fractional changes per time vs. fractional changes in power output over the different metabolic phases of the exercise test. Supplementary Figure 2 depicts the observed linear relationships between the increments for the main characteristics, i.e., VO_2_, VE, Q, DSpO_2_, DSmO_2_ in *m. vastus lateralis*, and PO.

The steepest slopes (i.e., highest control coefficients) resolved for VE (1.00), DSmO_2_ (0.061), with lower values for Q (0.032), DSpO_2_ (0.005), and VO_2_ (0.004). Control coefficients for aspects of the pathway of oxygen vs. VO_2_ were essentially identical to those observed vs. PO.

Few associations with aerobic fitness and/or the ACE-I/D genotype were resolved for the control coefficients of the parameters of the oxygen pathway (VO_2_, VE, Q, DSPO_2_, DSmO_2_ Vas, DSmO_2_ Gas, THb Vas, THb Gas, RER; Supplementary Table 3).

For aerobic fitness, this concerned the power-related slopes for increments of THb Vas (h2 = 0.183, *p* = 0.005). For the genotype this concerned DSmO_2_ (h2 = 0.117, *p* = 0.027), and at the level of a trend THb in *m. vastus lateralis* (h2 = 0.069, *p* = 0.093) and SpO_2_ (h2 = 0.096, *p* = 0.055). For the interaction between fitness state × genotype the associations concerned the power-related slopes for increments SpO_2_ (h2 = 0.089, *p* = 0.076).

The slope of the relationship between the increment of THb in *m. vastus lateralis* vs. power, alone, demonstrated an association with the sex (*p* = 0.007), which disappeared when the interaction with the aerobic fitness state (*p* = 0.841) and/or ACE-I/D genotype (*p* = 0.230) was considered.

At the *post-hoc* level, these differences localized to higher power-related control coefficients for THb in *m. vastus lateralis* in aerobically fit than unfit subjects (**Figure 5**). For the deficit in SpO_2_ and SmO_2_ higher control coefficients revealed for fit D-allele carriers than fit non-carriers. Conversely, the respective control coefficient for THb in *m. vastus lateralis* was lower in unfit non-carriers than unfit carriers of the D-allele, and fit non-carriers of the D-allele. Sex did not exert a significant influence on aerobic fitness, and ACE-I/D genotype associated effects on the control coefficients vs. power output.

## Discussion

Muscle work importantly elevates metabolic rates, whereby the ratio between metabolic and mechanical work is on average around one to four (Kyrolainen et al., 1995; Weibel and Hoppeler, 2005). There is a considerable inter-individual variability in this metabolic efficiency (Williams et al., 2004). In absence of a clearly identifiable single gatekeeper, potentially, this has its roots in different capacities and exercise-induced regulation of any of the connected metabolic steps of the systemic pathway of oxygen. For instance, resting and activity-induced rates of aerobic metabolism, and contraction-induced capillary perfusion demonstrate considerable inter-individual differences, which are associated with aerobic fitness and genetic factors (York et al., 1980; Zurlo et al., 1994; Donahoo et al., 2004; van Ginkel et al., 2016). To understand the underpinning metabolic aspects, we carried out a first investigation characterizing linear relationships between power output and parameters that are part of the systemic pathway of aerobic metabolism during a test of ramp exercise. The hypothesis being that the power-related slopes for raw values during the work phase, and the respective rates during work and rest phases of the ramp test (as a measure of the control coefficients) would differ in association with the state of aerobic fitness and the ACE-I/D genotype (Suarez and Moyes, 2012). The calculated high correlation coefficients highlight the considerable coupling between the systemic aspects of aerobic metabolism and power output (Supplementary Figure 1). Differences in the slopes of respective linear relationships of VO_2_, VE, and RER vs. PO confirm that the better metabolic efficiency of aerobically fit subjects during exercise at peak intensity involves an increased contribution of aerobic metabolism to the generation of muscle power. Importantly, the reliance of power output on the former-mentioned three inter-related systemic aspects of aerobic metabolism, as well as oxygen saturation in *m. vastus lateralis* was higher in fit than unfit carriers of the D-allele. Collectively, our findings are in line with the reported interacting associations of fitness state × ACE-I/D genotype on the development of mitochondrial muscle metabolism (Valdivieso et al., 2017).

**Figure 4:**
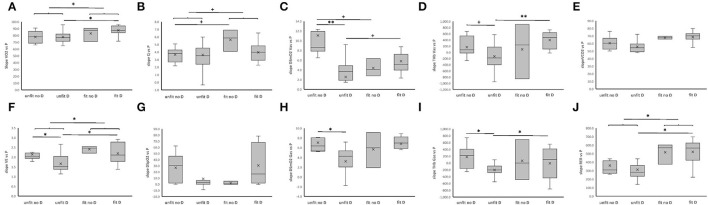
ACE-I/D genotype and aerobic fitness associated differences in power-related linear relationships of aerobic metabolism during pedaling. **(A–J)** Box-Whisker plots for the slope of linear relationships between parameters of the pathway of oxygen and power output during the work phase of the ramp exercise test. Box, first and third quartile; central line, median; x, mean; whisker, minima-maxima. *p < 0.05 for the indicated comparison. ANOVA with *post-hoc* test of Fisher. ^+^, *, **Indicate *p* < 0.10, <0.05 and <0.001, respectively, for the indicated comparison. Swung brackets refer to data from a same fitness state. ANOVA with *post-hoc* test of least significant difference.

The higher values for the slope of relationship for VO_2_ and VE, and Q (*p* = 0.076), respective to power output during muscle work in fit subjects, imply that the better metabolic efficiency involved an increased contribution, and coupling, of cardio-pulmonary function to the skeletal muscle's contractile function. These effects were related to steeper power-related slopes of RER (Figure 4) which stand for a higher rate of carbohydrate consumption (van Ginkel et al., 2015; Hall and Hall). Indeed, the former effects were paralleled by fitness-related differences in the concentration of blood glucose during the work phase, but only in the non-carriers of the D-allele (Figure 2). This emphasizes that an increased aerobic usage of carbohydrates at the muscle level has contributed, together with possibly other factors, such as fiber type composition (Coyle et al., 1992; Woods et al., 2002; Fluck et al., 2019; Hall and Hall), to improve the metabolic efficiency of the fit subjects at the maximal intensity of exercise when voluntary exhaustion manifested (Woods et al., 2002; van Ginkel et al., 2015; Fluck et al., 2019; Hall and Hall) Intriguingly, the power-related slope of DSmO_2_ in *m. vastus lateralis*, which is reflective of the respiratory capacity of this knee extensor muscle (Ryan et al., 2014), was not associated with the fitness state, alone (*p* = 0.393). However, when the ACE-I/D genotype was taken into consideration, a fitness-related association was identified for DSmO_2_. At the *post-hoc* level, this resolved to a lower reliance of power output on DSmO_2_ for the unfit carriers compared to non-carriers of the D-allele, and a trend for an increased dependency on DSmO_2_ for fit compared to unfit non-carriers of the D-allele. Further, we observed similar fitness × genotype associations for the power-related slopes of DSmO_2_ in *m. gastrocnemius*, as well as THb in both muscles (Figure 4). These connected observations highlight that the ACE-I/D genotype affected the influence of the fitness state on the contribution of oxygen transport and its use in recruited leg muscle groups to power production. A similar observation was made for VE, supporting the idea that fitness-state associated differences in activity-induced aerobic metabolic processes are influenced at the muscle and pulmonary level by the ACE-I/D genotype. Thereby, the observed control coefficients respective to power output decreased in the order of VE ≥ DSmO_2_ ≥ Q, indicating that VE demonstrated the hierarchically most important aspect that influenced PO (Suarez and Moyes, 2012).

**Figure 5:**
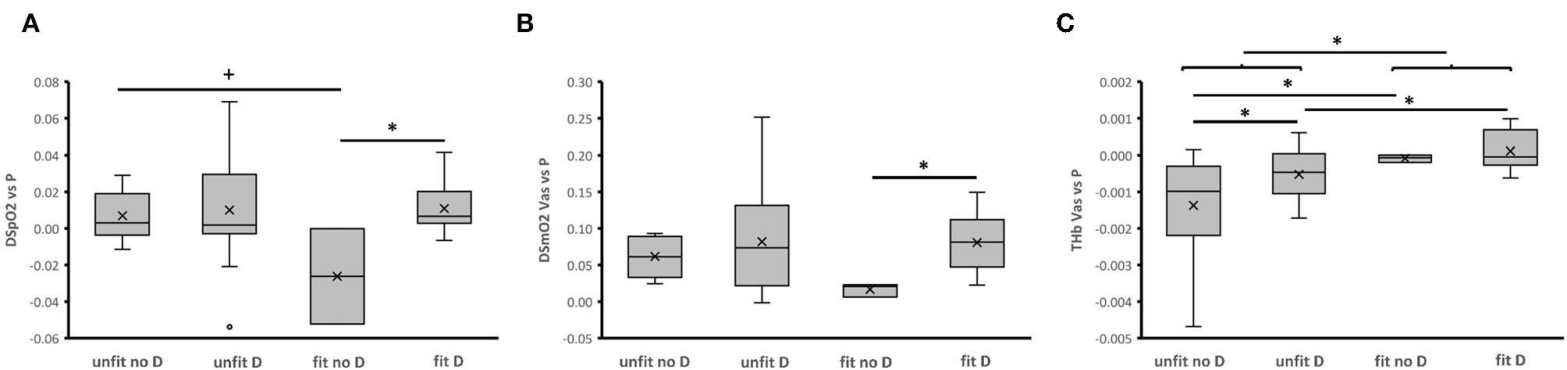
ACE-I/D genotype and aerobic fitness associated differences in the control coefficients for aspects of aerobic metabolism. Box-Whisker plots of the fitness- and ACE-I/D-genotype dependent control coefficients for DSpO_2_ **(A)**, DSmO_2_ Vas **(B)**, and THb Vas **(C)** respective to power output during the different metabolic phases of the ramp exercise test. Box, first and third quartile; central line, median; x, mean; whisker, minima-maxima. ^+^, *Indicate *p* < 0.10 and <0.05, respectively, for the indicated comparison. Swung brackets refer to data from a same fitness state. ANOVA with *post-hoc* test of least significant difference.

Differences in the control coefficients for DSmO_2_ and THb vs. PO over all phases of the ramp test, including the transition from rest to exercise and vice-versa (Figure 5), support the notion that inter-individual differences in the reliance of power output on oxygenation and perfusion of *m. vastus lateralis* are associated with the interaction between the ACE-I/D genotype with fitness state. However, due to a genotype × fitness interaction on energy expenditure at rest (Table 2) the resulting trends and significance levels were at variance. Additionally, we noted a trend for an interaction between the ACE-I/D genotype and fitness state for the control coefficient of SpO_2_ (h2 = 0.089, *p* = 0.076). Collectively, the findings corroborate the view that muscle-dependent production of power in the fit ACE D-allele carriers, relied more on aspects of aerobic metabolism than in the fit non-carriers of the D-allele.

The precipitous drop of muscle oxygen saturation with contraction results from an enhanced oxygen consumption in mitochondria which is not fully matched by the increase in muscle perfusion (Özyener, 2002; Jones et al., 2017). Measurements of the deficit in SmO_2_ allow an estimate of the rate of mitochondrial respiration in skeletal muscle (Ryan et al., 2014). This capacity for local aerobic metabolism is largely set by the volume density of mitochondria (Hoppeler and Weibel, 2000). Consequently, we interpret our observations on the lower control coefficient for DmSO_2_ respective to power output in fit non-carriers respective to non-carriers of the D-allele (Figure 5), and the conversely higher power-related slopes in unfit non-carriers respective to carriers of the D-allele (Figure 4), as to relate to differences in mitochondrial volume density (Figure 5). In this respect it is relevant that the ACE-I/D gene polymorphisms are associated with differences in gains in mitochondrial volume densities, in *m. vastus lateralis* with endurance training (Vaughan et al., 2013; Fluck et al., 2019). It remains to be elucidated to which extent the indicated higher reliance of *m. vastus lateralis* from the fit D-allele non-carriers on mitochondrial metabolism, through an expected higher volume density of mitochondria, translates into a more economic energy supply to the working muscle compared to the fit D-allele carriers. The former notion is supported by the observed power-related slopes for DSpO_2_, which is a function of cardiovascular delivery and removal of oxygen, whereby mitochondrial respiration is the most important, single contributor (Korzeniewski and Rossiter, 2015). Accordingly, the higher control coefficients of DSpO_2_ for the fit carriers than fit non-carriers of the D-allele resembled the observed differences for DSmO_2_ (Figure 5). As well, in fit compared to unfit D-allele carriers we identified lower values for the concentration of blood lactate during the work phase of the ramp test (Figure 2). This observation indicates a reduced strain in the oxygen-dependent metabolization of glucose-derived pyruvate in muscle mitochondria. Correspondingly, we observed a trend for a higher reliance of PO on DSmO_2_ in the fit compared to unfit D-allele carriers during muscle work (Figure 4). Overall, our findings imply that mitochondrial respiration rates in recruited muscle groups represented a relatively more important contributor for increasing power output in D-allele carriers than non-carriers for the aerobically fit subjects.

The observed influence of the fitness state and possibly ACE-I/D genotype on the estimated control coefficients for rates of change in THb respective to the produced power indicates that muscle perfusion may also importantly modulate muscle performance. Steady-state rates of muscle perfusion, as being reflected by THb in skeletal muscle, are known to be affected with the onset of muscle work, due to the interplay of contraction-induced vasodilatation and higher intra-muscular pressures acting on, and possibly compressing blood vessels during contraction (Gasser et al., 2021), even during dynamic exercise (Joyner and Casey, 2015). In fact, the overall negative control coefficients for THb vs. power (Supplementary Figure 2) emphasize that the rate changes for THb concentration vs. those of power were effectively negative during the work phase while being increased during recovery. The suggested lowering of THb, and consequently perfusion of *m. vastus lateralis* during pedaling, effectively manifested in negative power-related slopes for THb in the unfit D-allele carriers (Figure 4). This observation is in line with the reportedly reduced capillary perfusion in D-allele carriers with exercise (van Ginkel et al., 2015, 2016) which is related to higher blood concentrations of the vasoconstrictory angiotensin 2 peptide (Korthuis, 2011; Valdivieso et al., 2017). Additionally, to this genetic influence on regulative aspects of capillary perfusion, capillary density of *m. vastus lateralis* that sets the capacity for muscle perfusion, demonstrates higher values for not-specifically trained ACE-II genotypes than ACE-ID and –DD genotypes (Valdivieso et al., 2017). The findings corroborate the view that, especially in unfit subjects, hemoglobin-mediated oxygen delivery and mitochondrial oxygen consumption increasingly contributed to the fueling of knee extensor and ankle extensor muscle contraction for non-carriers compared to carriers of the D-allele (Tonkonogi and Sahlin, 2002; Zoll et al., 2002; Clifford and Hellsten, 2004).

Our findings also imply that the interdependencies between muscle perfusion and muscle respiration are specifically more important during the recovery phase, when the rate of mitochondrial oxygen consumption is expected to be level off (Korzeniewski and Rossiter, 2015). Especially, the observed variance in control coefficients for THb with the fitness state, and the *post-hoc* influence of the ACE-I/D-genotype (Figure 5), highlights the importance of muscle perfusion for aerobic fitness and supports that the ACE-I/D genotype modulates this influence. These observations possibly explain part of the observed inter-subject variability in exercise-induced elevations of muscle perfusion (Garten et al., 2014), which can in fractions be attributed to differences in aerobic capacity (Joyner and Casey, 2015).

Intriguingly, and despite former findings (Williams et al., 2017), aerobic capacity (as set by absolute and body mass-related peakVO_2_) was not identified to be associated with the ACE-I/D genotype (*p* = 0.475), but body mass and the related BMI were affected (Table 1). Possibly this indicates the limited, contribution of single heritable factors on the multifactorial pathway that set whole-body oxygen uptake and metabolism (Hoppeler, 2018). Furthermore, there might be an effect of age as it is known that for example sarcopenia is clearly an age-related phenomenon (Santos and Sardinha, 2017). Body mass index has been reported before to be associated with the ACE I/D polymorphism, whereby, the I-allele was more frequent in subjects demonstrating a BMI in excess of 30 kg m^2^ (Wacker et al., 2008). We have noted before that the cross-sectional area of the entire *m. vastus lateralis* and embedded slow-type muscle fibers are higher in ACE I allele carriers (Valdivieso et al., 2017). This suggests that the identified association of the ACE-I/D genotype, in interaction with the fitness state, on maximal oxygen uptake (Flueck et al., 2010) may reflect an influence of (lean) body mass on metabolic rate, through the in-here identified effects on DSmO_2_ during muscle work (reviewed Hopker et al., 2010). As well, based on the assessed slopes of relationships to power (Table 4; Figure 4), we only identified a trend for an ACE-I/D genotype-related influence on cardiac aspects of endurance performance (Jones et al., 2002; Hernández et al., 2003; Puthucheary et al., 2011). Overall, the ACE-I/D genotype associated influence of the fitness state on power-related dependencies of VE, RER, SmO_2_, and THb during exercise, relates to the superior trainability of aerobic fitness in ACE-II genotypes (Flueck et al., 2010). The present novel observations, therefore, imply that the reported influence of the ACE-I/D genotype on metabolic efficiency and VO_2_max (Weibel and Hoppeler, 2005; Puthucheary et al., 2011; Hoppeler, 2018) is related to a combination of pulmonary and muscular aspects of aerobic metabolism.

The identified effects should be viewed in terms of the limitations of our study. The conducted analyses encompass a relatively small sample. This explains by the prospective calculation of the required sample size to reach statistical significance of the hypothesized interaction effects between ACE-I/D genotype and aerobic fitness, as well as by budget constraints and the fact, that it was difficult to motivate participants to participate in for the study. Furthermore, limitations arise due to the experimental design where the distinction between fit and unfit subjects was solely based on standardized measures of VO2peak in a single test of exhaustive ramp exercise. A sensitivity analysis (Supplementary Table 1) indicates that main effects and most post-hoc effects are preserved when applying age-specific cut-off values for VO_2_ peak from various healthy western population to define the state of aerobic fitness. Accordingly, we mapped a number of effects that are consistent with the literature. First, we report considerable reductions in the computed levels of muscle oxygen saturation during the task of exhaustive exercise, which are in line with the reported larger degrees for muscle deoxygenation and re-oxygenation between the aerobically fit and unfit subjects (Montgomery and Brull, 2000; Brizendine et al., 2013). Importantly for SmO_2_ that is understood to reflect the rate of mitochondrial respiration in skeletal muscle (Ryan et al., 2014), we found in consistence with previous findings a high level of linear relationship to the values of VO_2_ (Figure 2; Zoladz et al., 1995; Boone et al., 2016). Second, we studied male and female subjects combined, because we did not identify a significant influence of this factor on the association of the characteristics of aerobic capacity and performance with the aerobic fitness state and/or ACE-I/D genotype. We also emphasize that fractional changes per time for the assessed parameters only provide estimates of metabolic flux, especially because values were compared for short periods of a ramp protocol, which individually do not represent stable metabolic steady states. The consequent influence may be critical for certain measured factors that are subject to subtle regulation. For instance, we noted that the values for SpO_2_ fluctuated considerably during periods when subjects were not pedaling against a resistance, possibly reflecting the reported patterns of alterations in human subjects in a metabolically unchallenged state, when blood flow is low (Korzeniewski and Rossiter, 2015). As oxygen saturation reflects only the difference between oxygen supply and demand changes during exercise are potentially larger (Rosenberry et al., 2019). To come over this limiting fact, we carried out the ramp exercise in the mere absence of a warm-up, respectively no activation of mitochondrial respiration, nevertheless some bias remains (Nioka et al., 1998; Boone et al., 2012; Perrey and Ferrari, 2018).

## Conclusive Summary

Using a vertical approach focusing on the influence of a maximal, exhaustive effort, we resolve that interactions between the ACE-I/D genotype × aerobic fitness are associated with variability in the contribution of pulmonary, cardiovascular, and muscle aspects of aerobic metabolism to work production by contracting muscle. Thereby the deployed method of verifying linear relationships appears useful to qualify the relative taxing of elements of the pathway of oxygen during a specific exercise challenge and to identify elements of systemic aerobic metabolism, which may limit the individual capacity for endurance-type sports.

The results motivate a model where inter-individual differences in aerobic fitness and metabolic efficiency are explained by interacting influences of the ACE-I/D genotype on the oxygen transport system. Thereby relevance is granted from the perspective that the ACE-I/D genotype is the prototype of a prominent genetic variant being associated with muscle performance, where the I-allele is reported to be enriched in subjects with an improved trainability of muscle-based performance exceeding efforts beyond a few minutes of continuous, intense exercise (Flueck et al., 2010). Our findings are in line with previously reported influences of the ACE-I/D genotype on maximal whole-body aerobic metabolisms, but which did not resolve in all instances (Flueck et al., 2010), due to very low effect sizes for VO_2_peak (0.009) and the contribution of VO_2_ (0.013, 0.013) and cardiac output (0.043, 0.040) to the produced power, irrespective of whether genotype alone, or the interaction with the fitness state was considered. Compared to the former observations, the in-here identified effect sizes for a genotype-associated contribution of minute ventilation (0.100), and genotype × fitness associated contribution of a deficit in muscle oxygen saturation (0.136) and muscle hemoglobin concentration (0.090), to the produced muscle power were larger. Consequently, our observations advocate a distinct fitness-related contribution of the ACE-I/D genotype on the efficiency, and possibly economy, of aerobic metabolism, and its fatigue resistance, to fuel cycling muscle contractions.

## Data Availability Statement

The datasets presented in this study can be found in online repositories. The names of the repository/repositories and accession number(s) can be found in the article/Supplementary Material.

## Ethics Statement

The studies involving human participants were reviewed and approved by Ethics Committee of Cantone Zuerich. The patients/participants provided their written informed consent to participate in this study.

## Author Contributions

MF, AF, DN, and WF designed the study. MF contributed to funding. MF, AF, SC, and WF performed experiments. MF, AF, and SC analyzed experiments. MF and AF analyzed the data. MF, DN, and BG interpreted the results. MF and BG drafted the manuscript and revised the manuscript. BG, AF, SC, DN, WF, and MF endorsed the manuscript. All authors contributed to the article and approved the submitted version.

## Funding

This study was supported by the Swiss Heart Foundation and the RESORTHO Foundation.

## Conflict of Interest

The authors declare that the research was conducted in the absence of any commercial or financial relationships that could be construed as a potential conflict of interest.

## Publisher's Note

All claims expressed in this article are solely those of the authors and do not necessarily represent those of their affiliated organizations, or those of the publisher, the editors and the reviewers. Any product that may be evaluated in this article, or claim that may be made by its manufacturer, is not guaranteed or endorsed by the publisher.

## Supplementary Material

Supplementary Figure 1 Network of correlations. Line graph showing the linear relationships surpassing a threshold of |*r*| > 0.65 and *p* < 0.05 for the average of comparisons over the studied subjects. Positive and negative correlations are depicted as solid and stippled lines, respectively. The number of correlations (connectivity) is given by the shading of each node where black and light gray corresponds to the highest and lowest connectivity. Note the high interactivity of linear relationships with PO.

Supplementary Figure 2 Control analysis—Line graphs visualizing the linear relationships between fractional changes per time for parameters of the pathway of oxygen and fractional changes in PO over the different metabolic phases of the ramp exercise test. Circles and vertical lines indicate the median + SE for the different parameters in the metabolic phases (rest, work, recovery, and transitions in between). Only values of parameters were shown which were resolved at the selected scale. Red and blue zones indicate exercise (red) and recovery (blue) phases of the exercise test. *n* = 44.

Supplementary Table 1 Sensitivity analysis on the influence of threshold of fitness on statistical sizes. Values refer to p-values, effect sizes and statistical power for main effect of the factors genotype (D-allele, no D-allele) × fitness (fit, unfit) on power-related slopes for the given parameters. Multifactorial ANOVA. Fitness was calculated with different method based on reference values. Thld50: Threshold of 50 mL O_2_ min^−1^ kg^−1^ according to the American Society of Sportsmedicine (7,31). Mscnd: mean of Scandinavian reference values (62), Mscnd_pol: interpolated mean of Scandinavian reference values (62), Nval: Norm values for VO_2_max (63), Mscnd50: Mean of Scandinavian reference values and minima of 50 mL O_2_ min^−1^ kg^−1^ (63), QDE: Upper quantile in Germany (64), CalC: Calculated values (65). Bold values indicate effects which were deemed significant.

Supplementary Table 2 Prospective power analysis. Calculation of the prospectively required number of biological replica to reveal statistically significant associations.

Supplementary Table 3 Association of fitness state × genotype for control coefficients characterizing the pathway of oxygen. List of the significance (*p*) and effect size (h2) of the interaction effects between aerobic fitness × ACE-I/D genotype on the control coefficients for the parameters of the pathway of oxygen and power output during the metabolic phases of the exercise test. ANOVA. Bold underlined values met the criteria of *p* < 0.05. *N* = 44.

## Abbreviations

ACE: angiotensin-converting enzyme
ACE-I/D: insertion/deletion polymorphism in the ACE gene
CC: Control coefficient
DSmO_2_: increasing deficit in muscle oxygen saturation
DSmO_2_ Vas: DSmO_2_ in *m. vastus lateralis*
DSmO_2_ Gas: DSmO_2_ in *m. gastrocnemius*
DSpO_2_: increasing deficit in arterial oxygen saturation energy
Erest: Energy expenditure during the rest phase
*m*.: *Musculus*
PO: power output
PPO: peak power output (W)
relative VO_2_peak: body-mass related VO_2_peak (mL O_2_ min^−1^ kg^−1^)
RER: Respiration exchange ratio
RERrest: RER during the rest phase
SmO_2_: muscle oxygen saturation
SpO_2_: arterial oxygen saturation
THb: total hemoglobin concentration (g dL^−1^)
THb Vas: THb in *m. vastus lateralis*
THb Gas: THb in *m. gastrocnemius*
VCO_2_: carbon dioxide consumption (L O_2_ min^−1^)
VE: minute ventilation in (L min^−1^)
VO_2_: oxygen uptake (L O_2_ min^−1^)
VO_2_peak: peak oxygen uptake (mL O_2_ min^−1^)
VO_2_max: maximal oxygen uptake
VO_2_rest: VO_2_ during the rest phase (mL O_2_ min^−1^)
Q: cardiac output in equivalents of L min^−1^.
