# Supplementary figures and images for: Variability in the Aerobic Fitness-Related Dependence on Respiratory Processes During Muscle Work Is Associated With the ACE-I/D Genotype
Source: Front Sports Act Living. 2022 May 19;4:814974. doi: 10.3389/fspor.2022.814974 (PMC9161700; doi:10.3389/fspor.2022.814974)

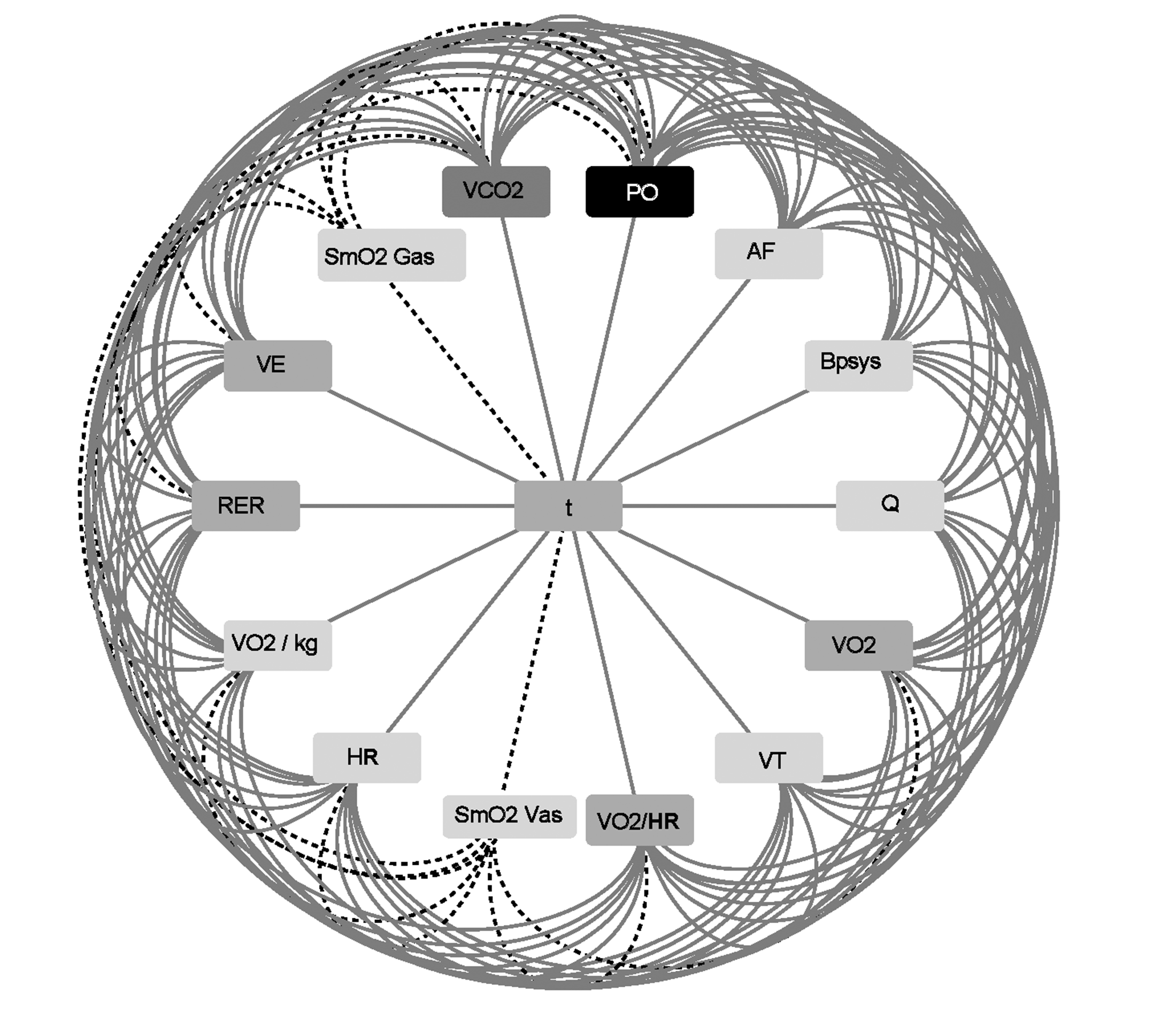

Supplement: Supplementary Figure 1 — Network of correlations. Line graph showing the linear relationships surpassing a threshold of |r| > 0.65 and p < 0.05 for the average of comparisons over the studied subjects. Positive and negative correlations are depicted as solid and stippled lines, respectively. The number of correlations (connectivity) is given by the shading of each node where black and light gray corresponds to the highest and lowest connectivity. Note the high interactivity of linear relationships with PO. [file Image_1.tif]

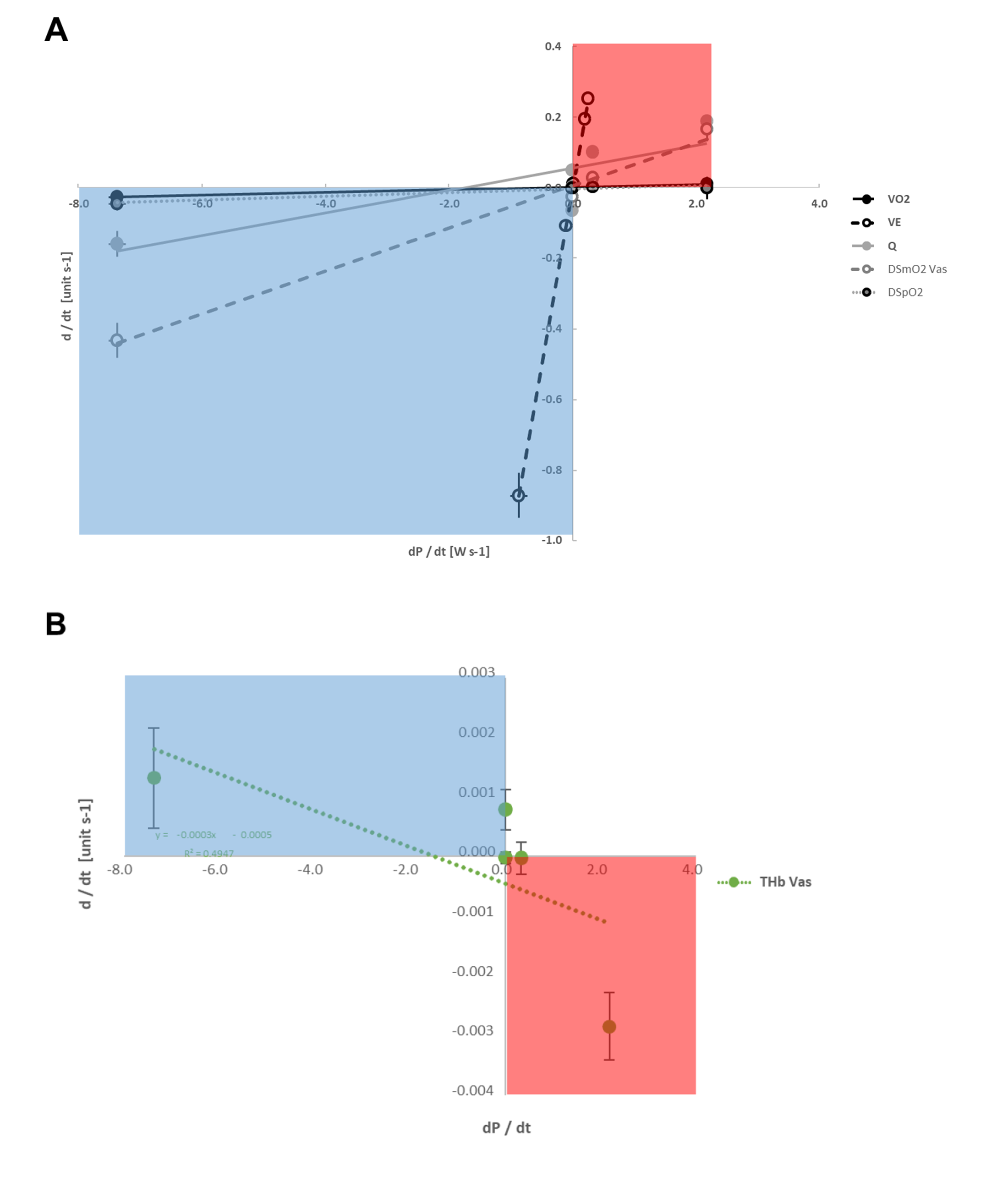

Supplement: Supplementary Figure 2 — Control analysis—Line graphs visualizing the linear relationships between fractional changes per time for parameters of the pathway of oxygen and fractional changes in PO over the different metabolic phases of the ramp exercise test. Circles and vertical lines indicate the median + SE for the different parameters in the metabolic phases (rest, work, recovery, and transitions in between). Only values of parameters were shown which were resolved at the selected scale. Red and blue zones indicate exercise (red) and recovery (blue) phases of the exercise test. n = 44. [file Image_2.tif]
