# Supplementary material for: Variability in the Aerobic Fitness-Related Dependence on Respiratory Processes During Muscle Work Is Associated With the ACE-I/D Genotype
Source: Front Sports Act Living. 2022 May 19;4:814974. doi: 10.3389/fspor.2022.814974 (PMC9161700; doi:10.3389/fspor.2022.814974)
Supplement: Supplementary Table 1 — Sensitivity analysis on the influence of threshold of fitness on statistical sizes. Values refer to p-values, effect sizes and statistical power for main effect of the factors genotype (D-allele, no D-allele) × fitness (fit, unfit) on power-related slopes for the given parameters. Multifactorial ANOVA. Fitness was calculated with different method based on reference values. Thld50: Threshold of 50 mL O2 min−1 kg−1 according to the American Society of Sportsmedicine (7,31). Mscnd: mean of Scandinavian reference values (62), Mscnd_pol: interpolated mean of Scandinavian reference values (62), Nval: Norm values for VO2max (63), Mscnd50: Mean of Scandinavian reference values and minima of 50 mL O2 min−1 kg−1 (63), QDE: Upper quantile in Germany (64), CalC: Calculated values (65). Bold values indicate effects which were deemed significant. [file Table_1.docx]

**Supplemental table 1:** *Sensitivity analysis on the influence of threshold of fitness on statistical sizes.* Values refer to p-values, effect sizes and statistical power for main effect of the factors genotype (D-allele, no D-allele) x fitness (fit, unfit) on power-related slopes for the given parameters. Multifactorial ANOVA. Fitness was calculated with different method based on reference values. Thld50: Threshold of 50 mL O2 min-1 kg-1 according to the American Society of Sportsmedicine (7,31). Mscnd: mean of Scandinavian reference values (62), Mscnd_pol: interpolated mean of Scandinavian reference values (62), Nval: Norm values for VO2max (63), Mscnd50: Mean of Scandinavian reference values and minima of 50 mL O2 min-1 kg-1 (63), QDE: Upper quantile in Germany (64), CalC: Calculated values (65). Bold values indicate effects which were deemed significant.

**p-value effect size power**

***Factor Parameter Thld50 Nval QDE Mscnd Mscnd_pol Mscnd50 CalC Thld50 Nval QDE Mscnd Mscnd_pol Mscnd50 CalC Thld50 Nval QDE Mscnd Mscnd_pol Mscnd50 CalC***

**fitness** **VO2** **0.0218** **0.0103** **0.0204** 0.0931 **0.0413** **0.0155** 0.1050 0.1247 0.1534 0.1272 0.0689 0.1001 0.1377 0.0644 0.6443 0.7475 0.6540 0.3896 0.5392 0.6938 0.3668

**VE** **0.0206** **0.0172** **0.0111** **0.0324** **0.0228** **0.0129** 0.0622 0.1268 0.1338 0.1507 0.1095 0.1229 0.1450 0.0842 0.6526 0.6792 0.7390 0.5808 0.6370 0.7196 0.4651

**Q** 0.0757 0.3368 0.5518 0.5589 0.4956 0.2326 0.1934 0.0768 0.0231 0.0089 0.0086 0.0117 0.0354 0.0419 0.4287 0.1578 0.0902 0.0887 0.1030 0.2194 0.2524

**SpO2** 0.8010 0.3249 0.3311 0.4771 0.3016 0.3068 0.3864 0.0017 0.0262 0.0255 0.0138 0.0288 0.0282 0.0203 0.0570 0.1633 0.1603 0.1077 0.1752 0.1725 0.1368

**SmO2Vas** 0.3926 0.2586 0.6972 0.9294 0.8037 0.7138 0.9364 0.0183 0.0318 0.0038 0.0002 0.0016 0.0034 0.0002 0.1347 0.2011 0.0669 0.0509 0.0569 0.0650 0.0507

**SmO2Gas** 0.4795 0.6361 0.6723 0.5753 0.5007 0.3726 0.8906 0.0126 0.0057 0.0045 0.0079 0.0114 0.0199 0.0005 0.1072 0.0752 0.0700 0.0855 0.1017 0.1424 0.0521

**THbVas** 0.1358 0.3964 0.1156 0.4497 0.2960 0.1409 0.1678 0.0548 0.0180 0.0608 0.0144 0.0273 0.0534 0.0470 0.3180 0.1333 0.3485 0.1157 0.1785 0.3111 0.2784

**THbGas** 0.7691 0.8218 0.8572 0.7876 0.8785 0.6846 0.6194 0.0022 0.0013 0.0008 0.0018 0.0006 0.0042 0.0062 0.0596 0.0556 0.0536 0.0581 0.0526 0.0685 0.0778

**RER** **0.0019** **0.0002** **0.0056** **0.0187** **0.0057** **0.0022** **0.0392** 0.2173 0.2924 0.1762 0.1306 0.1758 0.2117 0.1020 0.9017 0.9776 0.8144 0.6671 0.8134 0.8922 0.5480

**genotype** **VO2** 0.4654 0.6101 0.5685 0.5011 0.2805 0.4458 0.4283 0.0134 0.0066 0.0082 0.0114 0.0290 0.0146 0.0158 0.1111 0.0793 0.0868 0.1016 0.1874 0.1168 0.1223

**VE** **0.0409** **0.0123** **0.0068** **0.0301** 0.0746 **0.0305** **0.0200** 0.1004 0.1466 0.1694 0.1122 0.0773 0.1117 0.1281 0.5407 0.7252 0.7959 0.5928 0.4313 0.5907 0.6574

**Q** 0.2933 0.7471 0.8196 0.1888 0.3779 0.3818 0.8261 0.0276 0.0026 0.0013 0.0428 0.0195 0.0192 0.0012 0.1800 0.0616 0.0558 0.2567 0.1403 0.1388 0.0554

**SpO2** 0.8113 0.4181 0.4264 0.5233 0.5795 0.5710 0.4271 0.0016 0.0178 0.0172 0.0111 0.0084 0.0088 0.0171 0.0563 0.1255 0.1227 0.0962 0.0846 0.0862 0.1225

**SmO2Vas** 0.0795 **0.0092** **0.0296** **0.0165** **0.0137** **0.0070** 0.0594 0.0749 0.1578 0.1129 0.1353 0.1426 0.1682 0.0860 0.4193 0.7615 0.5954 0.6850 0.7114 0.7924 0.4736

**SmO2Gas** 0.3910 0.1117 0.1905 0.2027 0.3047 0.1789 0.2697 0.0185 0.0620 0.0425 0.0402 0.0263 0.0447 0.0304 0.1353 0.3550 0.2551 0.2439 0.1738 0.2666 0.1940

**THbVas** 0.9916 0.4315 0.3646 0.7878 0.9702 0.6768 0.3605 <0.0001 0.0155 0.0206 0.0018 <0.0001 0.0044 0.0209 0.0500 0.1213 0.1456 0.0581 0.0502 0.0695 0.1474

**THbGas** 0.1233 **0.0247** **0.0211** 0.0931 0.0810 0.0569 **0.0417** 0.0583 0.1198 0.1259 0.0689 0.0742 0.0877 0.0997 0.3362 0.6245 0.6489 0.3895 0.4159 0.4815 0.5375

**RER** 0.6946 0.5227 0.6106 0.7288 0.8734 0.8017 0.7849 0.0039 0.0103 0.0065 0.0030 0.0006 0.0016 0.0019 0.0672 0.0965 0.0792 0.0634 0.0528 0.0570 0.0583

**fitness x genotype** **VO2** 0.4757 0.9611 0.8047 0.8843 0.7033 0.9422 0.4335 0.0128 0.0001 0.0015 0.0005 0.0037 0.0001 0.0154 0.1082 0.0503 0.0568 0.0523 0.0662 0.0506 0.1206

**VE** 0.4305 0.4758 0.2799 0.8860 0.1900 0.8500 0.6005 0.0156 0.0128 0.0291 0.0005 0.0425 0.0009 0.0069 0.1216 0.1082 0.1877 0.0523 0.2556 0.0540 0.0809

**Q** 0.0705 **0.0289** **0.0221** 0.2016 0.1546 0.0297 0.1573 0.0795 0.1137 0.1242 0.0404 0.0500 0.1127 0.0494 0.4420 0.5992 0.6420 0.2448 0.2936 0.5949 0.2905

**SpO2** 0.0741 0.5009 0.7300 0.7027 0.9473 0.4820 0.7889 0.0837 0.0123 0.0033 0.0040 0.0001 0.0135 0.0020 0.4328 0.1016 0.0633 0.0662 0.0505 0.1064 0.0579

**SmO2Vas** **0.0163** 0.4030 0.4439 0.1044 0.1977 **0.0204** 0.3044 0.1359 0.0175 0.0147 0.0646 0.0411 0.1273 0.0263 0.6873 0.1309 0.1174 0.3678 0.2484 0.6545 0.1739

**SmO2Gas** 0.1153 0.0924 0.8178 0.9436 0.0840 0.1103 0.9632 0.0609 0.0692 0.0013 0.0001 0.0728 0.0625 0.0001 0.3490 0.3910 0.0559 0.0506 0.4089 0.3573 0.0502

**THbVas** 0.0534 0.1991 0.4239 0.2785 0.0862 0.1146 0.5162 0.0902 0.0409 0.0161 0.0293 0.0718 0.0611 0.0106 0.4933 0.2472 0.1237 0.1886 0.4042 0.3501 0.0980

**THbGas** 0.2970 0.1230 0.1841 0.1617 0.1207 0.1076 0.3492 0.0272 0.0584 0.0437 0.0484 0.0591 0.0634 0.0219 0.1780 0.3367 0.2614 0.2852 0.3403 0.3621 0.1522

**RER** 0.6238 0.9525 0.9389 0.9290 0.8741 0.6768 0.6658 0.0061 0.0001 0.0001 0.0002 0.0006 0.0044 0.0047 0.0771 0.0504 0.0507 0.0509 0.0528 0.0695 0.0709

**model** **VO2** **0.0093** **0.0323** 0.0843 0.1692 0.0634 **0.0392** 0.3139 0.2474 0.1950 0.1513 0.1170 0.1646 0.1865 0.0840 0.8375 0.6999 0.5525 0.4251 0.5998 0.6731 0.3024

**VE** **0.0020** **0.0054** **0.0027** **0.0163** **0.0015** **0.0105** **0.0453** 0.3061 0.2691 0.2954 0.2243 0.3162 0.2426 0.1800 0.9339 0.8801 0.9206 0.7827 0.9448 0.8269 0.6521

**Q** 0.2564 0.1457 0.1145 0.2818 0.4623 0.1481 0.3226 0.0951 0.1245 0.1365 0.0900 0.0616 0.1237 0.0825 0.3433 0.4536 0.4982 0.3243 0.2234 0.4504 0.2969

**SpO2** 0.1656 0.3804 0.5942 0.8603 0.6192 0.3018 0.7237 0.1269 0.0787 0.0493 0.0199 0.0464 0.0928 0.0347 0.4278 0.2620 0.1708 0.0931 0.1624 0.3090 0.1301

**SmO2Vas** **0.0041** **0.0393** 0.0572 **0.0154** **0.0224** **0.0041** **0.0457** 0.2790 0.1864 0.1694 0.2268 0.2109 0.2791 0.1796 0.8968 0.6727 0.6163 0.7890 0.7464 0.8969 0.6507

**SmO2Gas** 0.0511 0.2049 0.5827 0.5513 0.0585 0.0502 0.6569 0.1746 0.1070 0.0470 0.0506 0.1684 0.1754 0.0390 0.6339 0.3878 0.1758 0.1872 0.6128 0.6366 0.1510

**THbVas** **0.0054** 0.2420 0.1905 0.2947 0.0601 **0.0299** 0.3772 0.2689 0.0982 0.1108 0.0875 0.1671 0.1985 0.0736 0.8798 0.3548 0.4021 0.3152 0.6084 0.7104 0.2651

**THbGas** 0.1095 0.0920 0.1287 0.1084 0.0638 0.0848 0.1932 0.1387 0.1471 0.1307 0.1392 0.1644 0.1510 0.1101 0.5063 0.5372 0.4768 0.5081 0.5989 0.5514 0.3993

**RER** **0.0010** **0.0009** **0.0278** 0.0602 **0.0150** **0.0106** 0.2244 0.3305 0.3354 0.2016 0.1670 0.2277 0.2419 0.1023 0.9579 0.9618 0.7198 0.6082 0.7915 0.8254 0.3699

**constant** **VO2** **<0.0001** **<0.0001** **<0.0001** **<0.0001** **<0.0001** **<0.0001** **<0.0001** 0.9853 0.9856 0.9826 0.9846 0.9860 0.9864 0.9798 1.0000 1.0000 1.0000 1.0000 1.0000 1.0000 1.0000

**VE** **<0.0001** **<0.0001** **<0.0001** **<0.0001** **<0.0001** **<0.0001** **<0.0001** 0.9426 0.9413 0.9335 0.9420 0.9508 0.9451 0.9176 1.0000 1.0000 1.0000 1.0000 1.0000 1.0000 1.0000

**Q** **<0.0001** **<0.0001** **<0.0001** **<0.0001** **<0.0001** **<0.0001** **<0.0001** 0.8279 0.8294 0.8142 0.8190 0.8223 0.8367 0.7781 1.0000 1.0000 1.0000 1.0000 1.0000 1.0000 1.0000

**SpO2** **0.0092** **0.0023** **0.0047** **0.0010** **0.0007** **0.0008** **0.0075** 0.1698 0.2254 0.1963 0.2562 0.2699 0.2628 0.1776 0.7638 0.8916 0.8334 0.9352 0.9496 0.9424 0.7860

**SmO2Vas** **<0.0001** **<0.0001** **<0.0001** **<0.0001** **<0.0001** **<0.0001** **<0.0001** 0.4743 0.5390 0.4893 0.5227 0.5521 0.5581 0.4886 1.0000 1.0000 1.0000 1.0000 1.0000 1.0000 1.0000

**SmO2Gas** **<0.0001** **<0.0001** **<0.0001** **<0.0001** **<0.0001** **<0.0001** **<0.0001** 0.5773 0.5828 0.5316 0.5804 0.6292 0.6176 0.5183 1.0000 1.0000 1.0000 1.0000 1.0000 1.0000 1.0000

**THbVas** 0.0830 0.2565 0.5048 0.1354 0.0628 0.1097 0.5761 0.0732 0.0321 0.0112 0.0549 0.0839 0.0627 0.0079 0.4112 0.2025 0.1007 0.3186 0.4634 0.3585 0.0853

**THbGas** 0.9239 0.9962 0.9690 0.9711 0.7633 0.9436 0.8428 0.0002 <0.0001 <0.0001 <0.0001 0.0023 0.0001 0.0010 0.0510 0.0500 0.0502 0.0501 0.0601 0.0506 0.0544

RER **<0.0001** **<0.0001** **<0.0001** **<0.0001** **<0.0001** **<0.0001** **<0.0001** 0.8591 0.8451 0.7906 0.8382 0.8541 0.8524 0.7604 1.0000 1.0000 1.0000 1.0000 1.0000 1.0000 1.0000
