# Supplementary material for: Variability in the Aerobic Fitness-Related Dependence on Respiratory Processes During Muscle Work Is Associated With the ACE-I/D Genotype
Source: Front Sports Act Living. 2022 May 19;4:814974. doi: 10.3389/fspor.2022.814974 (PMC9161700; doi:10.3389/fspor.2022.814974)
Supplement: Supplementary Table 2 — Prospective power analysis. Calculation of the prospectively required number of biological replica to reveal statistically significant associations. [file Table_2.docx]

**Supplemental table 2: *Prospective power analysis.*** Calculation of the prospectively required number of biological replica to reveal statistically significant associations

***parameter groups effect size (ACE-I/D genotype) prospective n references URL***

mitochondrial volume density unfit vs fit 0.849 15 Flück et al, 2019 https://www.ncbi.nlm.nih.gov/pmc/articles/PMC6518954/

mitochondrial volume density unfit vs fit ACE-II 1.494 6 Flück et al, 2019 https://www.ncbi.nlm.nih.gov/pmc/articles/PMC6518954/

mitochondrial volume density unfit vs fit ACE-ID 1.36 8 Flück et al, 2019 https://www.ncbi.nlm.nih.gov/pmc/articles/PMC6518954/

mitochondrial volume density unfit vs fit ACE-DD 1.00 10 Flück et al, 2019 https://www.ncbi.nlm.nih.gov/pmc/articles/PMC6518954/

total mitochondrial volume density unfit vs fit ACE-I allele 0.073 1240 Vaughan et al. 2013 https://www.ncbi.nlm.nih.gov/pmc/articles/PMC3677975/

subsarcolemmal mitochondrial volume density unfit vs fit ACE-I allele 0.152 292 Vaughan et al. 2013 https://www.ncbi.nlm.nih.gov/pmc/articles/PMC3677975/

subsarcolemmal mitochondrial volume density unfit vs fit ACE-I allele 0.314 70 Vaughan et al. 2013 https://www.ncbi.nlm.nih.gov/pmc/articles/PMC3677975/

VO2max unfit vs fit 0.652 24 Vaughan et al. 2016 https://www.ncbi.nlm.nih.gov/pmc/articles/PMC4794249/

VO2max kg-1 unfit vs fit 1.201 12 Vaughan et al. 2016 https://www.ncbi.nlm.nih.gov/pmc/articles/PMC4794249/
