# Supplementary material for: Variability in the Aerobic Fitness-Related Dependence on Respiratory Processes During Muscle Work Is Associated With the ACE-I/D Genotype
Source: Front Sports Act Living. 2022 May 19;4:814974. doi: 10.3389/fspor.2022.814974 (PMC9161700; doi:10.3389/fspor.2022.814974)
Supplement: Supplementary Table 3 — Association of fitness state × genotype for control coefficients characterizing the pathway of oxygen. List of the significance (p) and effect size (h2) of the interaction effects between aerobic fitness × ACE-I/D genotype on the control coefficients for the parameters of the pathway of oxygen and power output during the metabolic phases of the exercise test. ANOVA. Bold underlined values met the criteria of p < 0.05. N = 44. [file Table_3.docx]

***Supplemental table 3:*** *Association of fitness state x genotype for control coefficients characterising the pathway of oxygen.* List of the significance (p) and effect size (h2) of the interaction effects between aerobic fitness x ACE-I/D genotype on the control coefficients for the parameters of the pathway of oxygen and power output during the metabolic phases of the exercise test. ANOVA. Bold underlined values met the criteria of p<0.05. N=44.

***aerobic fitness genotype aerobic fitness x genotype***

***p-value h2 p-value h2 p-value h2***

VO_2_ 0.373 0.02 0.842 0.001 0.119 0.060

VE 0.608 0.007 0.664 0.005 0.916 0.000

Q 0.698 0.004 0.297 0.029 0.956 0.000

D SpO_2_ 0.107 0.069 0.055 0.096 0.089 0.076

D SmO_2_ Vas 0.213 0.038 **0.027** 0.117 0.238 0.035

DSmO_2_ Gas 0.359 0.021 0.500 0.011 0.459 0.014

THb Vas **0.005** 0.183 0.093 0.069 0.321 0.025

THb Gas 0.706 0.004 0.793 0.002 0.748 0.003

RER 0.617 0.006 0.829 0.001 0.245 0.034
